# Evolution of plant sex and molecular mechanisms underlying plants sex separation

**DOI:** 10.48130/FR-2023-0001

**Published:** 2023-01-13

**Authors:** Wei Li, Wei Fu, Jing Hou, Yonghua Yang, Tongming Yin

**Affiliations:** 1 College of Landscape and Horticulture, Yangzhou Polytechnic College, Yangzhou 225009, China; 2 Co-Innovation Center for Sustainable Forestry in Southern China, Key Laboratory of Tree Genetics and Biotechnology of Educational Department of China, Key Laboratory of Tree Genetics and Sivilcultural Sciences of Jiangsu Province, Nanjing Forestry University, Nanjing 210037, China; 3 College of Horticulture and Plant Protection, Yangzhou University, Yangzhou 225012, China; 4 Institute for Plant Molecular Biology, State Key Laboratory of Pharmaceutical Biotechnology, School of Life Sciences, Nanjing University, Nanjing 210023, China

**Keywords:** Plant sex, Dioecy, Sex chromosome, Sex-determining gene, Plant sex evolution

## Abstract

Unlike animals, plants exhibit more complexity of sexual morphs. The genetic mechanism underlying plant sex is a hot research topic in plant biology. In recent decades, advanced theories have been put forth on plant sex determination, but experimental proof is scarce. In recent years, vast achievements have been made to reveal the genetic mechanisms underlying sex separation of plants at the molecular level. Although the sex determination mechanisms have been clarified only in a limited number of plant species thus far, the discoveries offer us an opportunity to understand the genetic mechanisms triggering the separation of plant sexes. This paper reviewed the different aspects of the advanced studies on plant sex evolution and the molecular mechanisms underlying plant sex separation.

## Introduction

Flowers are the reproductive organ of flowering plants, classified as unisexual or bisexual flowers. The bisexual flower contains fertile stamen and pistil in one flower, while the stamen or pistil aborts in the process of flower development in unisexual flowers. Compared to animals, sexual morphs of flowering plants are more complex, with the majority being hermaphroditic, whose flowers have both male and female organs. The other extremities are the dioecious plants, which bear unisexual flowers of the alternate sexes on separate plants. There are some intermediate sexual morphs, including monoecy: plants bear unisexual flowers of the alternate sex; gynomonoecy: plants bear female and hermaphroditic flowers; andromonoecy: plants bear male and hermaphroditic flowers; gynodioecy: plants with individuals bear hermaphroditic flowers and individuals bear female flowers; androdioecy: plants with individuals bear hermaphroditic flowers and individuals bear male flowers^[1−5]^.

In plants, being hermaphrodite is considered as the most primitive sexual morph, and dioecy is the most advanced. In different phyla of botany, the proportion of dioecious species varies a lot. About 68% of mosses, 57% of *Marchantia polymorpha*, and 40% of *Anthocerotae* are dioecious in Bryophytes^[6]^. There is also a striking difference in spermatophytes: among the reported 1,033 gymnosperm species, 667 are dioecious^[7]^, whereas only about 5%−6% of the 300,000 species in angiosperm plants are dioecious^[8]^. In natural populations, it is possible that multiple sexual morphs are observed in a variety of flowering plants^[9]^. Sexual morphs of higher plants are determined by the joint effects of sex chromosomes, sex-related genes, phytohormone, and environmental influence. Sex-related genes in plants include sex-determining genes and sex differentiation genes. Sex-determining genes trigger the initiation of the development of the alternate sexual organs, while sex differentiation genes express afterward and are differentially expressed in different tissues, organs, and individuals, resulting in formation of flowers of different sexes^[10]^.

Bisexual flowers could either spread or receive pollen simultaneously. A set of identical attractions (petal, pollen, nectar etc) appeal to pollinators to carry and transmit pollen, which maximizes parent functions, saving resources and energy consumption. Nevertheless, this benefit also has retribution in reproduction, causing inbreeding depression and reduction in genetic diversity, especially the decline of genetic diversity. To resolve this, plants evolved dichogamy and heterogony, but the problem remains^[11]^. Together with self-incompatibility, not only dioecy but plants of other sexes except those with monoecy, produce unisexual flowers, which will more or less reduce or even avoid gender interference, thus obtaining heterosis and increasing the genetic diversity of the offspring^[12]^. That is part of the reasons for plant evolution. By summarizing the origin and evolution of plants from hermaphrodite to dioecy, pathways affecting sex determination and separation, and recent progress in studying sex determination, we propose some expectations for future research on sex determination in plants, such as expanding the sight from sex-determining genes to regulatory pathways, from a single species to relevant families and genera, and finally applying the identification technology in actual production and practice.

## The origin and evolution of plant sex chromosomes

**Figure 1:**
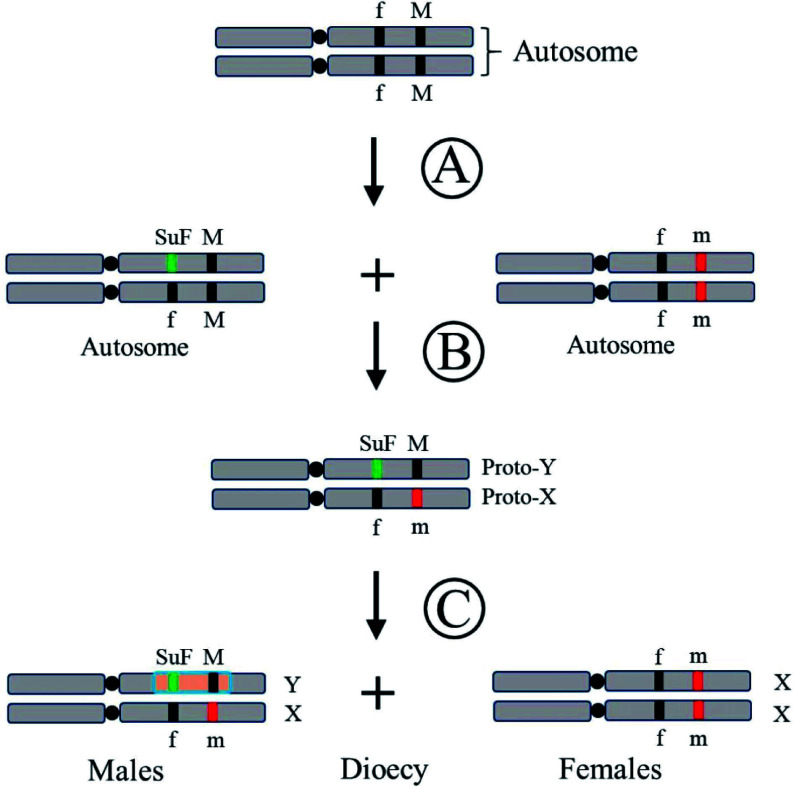
Sex determination system by the 'two mutations' model^[13]^. (A) One of a pair of autosomes has obtained a mutation of a certain sex sterility gene, '*M*' to '*m*' is a recessive mutation, '*f*' to '*SuF*' is a dominant mutation. (B) The other one autosome acquires the mutation corresponding to another sex sterility gene, thus generating proto-X and proto-Y, the precursor of sex chromosome. (C) Sterile genes are linked and sex chromosomes are preliminarily formed and dioecious plants emerged. The shaded part of the Y chromosome in (C) represents the sex-linked region.

Plant sex chromosomes have evolved multiple times from ancestors and through various evolutionary routes. It is generally accepted that unisexual flowers have evolved from hermaphroditic flowers through genetic mutations. Mutants are retained by random selection. Populations will then generate individuals with unisexual flowers and individuals with hermaphroditic flowers. Previous studies have discovered that two dominant complementary and closely linked genes first appeared in autosomes of the hermaphroditic ancestors: the dominant male fertile gene (*M*) and the recessive female suppressor gene (*suf*). There are two possible evolutionary pathways from hermaphrodite to dioecy^[13]^: one pathway is that the recessive *suf* first mutated into the dominant *SuF* to inhibit pistil development, thus producing unisexual male flowers, and then the dominant *M* mutated into the recessive *m* to produce unisexual female flowers; the second pathway is that the *M* locus first mutated to *m*, and then *suf* mutated to *SuF*, thereby generating dioecious plants. Mutation of gene *M* caused male sterility, subsequently, female dioecious plants evolved. With the loss of male fertility factors, pure female flowers gradually formed; Gene *suf* mutation inhibited pistil development, and then androdioecy developed (Fig. 1). With the enhancement of the female inhibitors, the pure male flowers evolved. When two infertility genes mutated, the recombination between the chromosomal mutation sites (sex-determining genes) was inhibited, causing the non-recombination region to gradually expand and eventually form heteromorphic sex chromosomes^[3]^.

At the early stages of the evolution of sex chromosomes, it is impossible to distinguish sex chromosomes from autosomes. Accumulated mutations of sex-determining genes cause the function change/loss of these genes, leading to the emergence of dioecy. In the process of evolution, sex chromosomes gradually diverged and eventually evolved into heteromorphic sex chromosomes, which had functional and morphological differences from autosomes. Some direct evidence in nature supports the theory of the evolution of sex chromosomes proposed by the above model. For example, the sex determination system of wild strawberries (*Fragaria virginana*) is the ZW sex chromosome system, in which, the female and male sterility genes were located in linkage group 6 with a genetic distance of 5.6 cM. Due to the recombination of these two genes, male, female, hermaphroditic, and neuter individuals are produced in the offspring. Nevertheless, sex chromosomes of *F. virginana* are still in the very early evolutionary stage, known as 'the incipient sex chromosomes'^[4]^.

**Figure 2:**
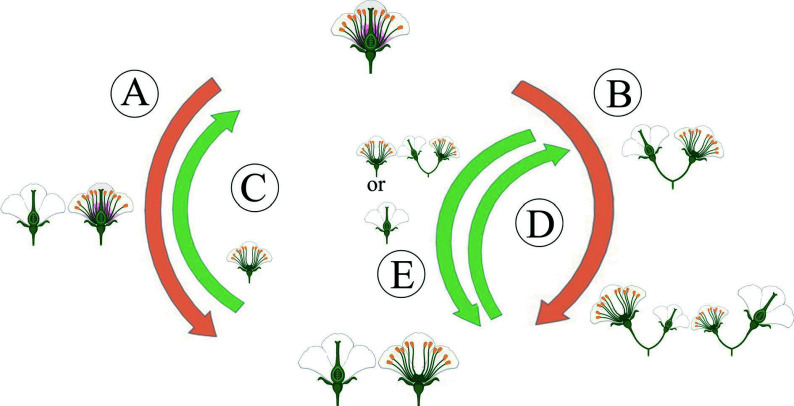
The main evolutionary routes to dioecy. (A) The gynodioecy-dioecy routes. (B) The monoecy-dioecy routes. (C), (D) It has been highlighted that dioecy may frequently revert to hermaphroditism. (C) In the gynodioecy-dioecy routes, inconstant males may help reversions to hermaphroditism. (D), (E) In the monoecy-dioecy route, there may be cycles between monoecy and dioecy.

Compared with other sexual morphs, dioecy has comprehensive advantages in reproduction, survival, and evolution. Moreover, dioecious plants have typical unisexual flowers^[9]^, which have probably not evolved directly from hermaphroditism, but indirectly from some intermediate pathways, including the monoecy-dioecy pathway, the gynodioecy-dioecy pathway, and the androdioecy-dioecy pathway^[14]^ (Fig. 2). Meanwhile, dioecious plants are desirable systems for studying the origin and evolutionary pathway of plant sex chromosomes. The monoecy-dioecy pathway is widespread among angiosperms, perhaps even more extensive than the gynodioecy-dioecy pathway^[8]^. The single-gene model has been used to illustrate the monoecy-dioecy pathway. Specifically, a single high-level regulator (a single gene) in the monoecious plant has two states (on *vs* off), allowing floral primordia to produce male or female flowers (monoecy), which could affect the flowers' spatial-temporal development. If this single gene was absent from some individuals, the male and female flowers separated on different individuals, leading the monoecy to evolve into dioecy^[15]^. Among the three pathways, the gynodioecy-dioecy pathway seems the most likely to occur in plants. In line with the two-mutations model described above, this presumed genetic model is that the male-promoting *M* locus mutated into the *m* locus, thus abolishing pollen production and hologyny plants evolved, leading to the coexistence of unisexual female (i.e., male-sterile individuals) and hermaphroditic individuals, which are called gynodioecy. Genetically, the gynodioecy-dioecy pathway and androdioecy-dioecy pathway are equivalent, but from the perspective of evolutionary ecology, pure female individuals do have advantages in reproduction due to the effect of inbreeding depression in hermaphrodites. However, for gynodioecious plants, the pollen of which must be transmitted to the stigma of bisexual plants, but it still has to compete with hermaphroditic pollens, i.e., it must produce more pollens than the pollinated plants to occupy the preponderance in the competition. Afterward, a dominant sex-linked mutation occurred in the gynodioecy population, resulting in male individuals. Consequently, the produced male and female individuals emerged as dioecy^[16]^. However, within the same genus, only herbaceous plants display gynodioecy, while woody plants do not, suggesting that low possibility of dioecy evolving from gynodioecy^[17]^. For the androdioecy-dioecy pathway, it first experienced a dominant mutation of ovary development in hermaphrodite plants, resulting in unisexual male plants (i.e., female-sterile individuals). Under this circumstance, male plants coexist with hermaphrodite plants, forming androdioecy. Although there is a striking rarity of androdioecy relative to gynodioecy, typical androdioecious systems have been found in *Phillyrea angustifolia*^[18]^ and *Mercurialis annua*^[14]^.

Notably, even the evolved dioecy is unstable. They can reverse into hermaphrodites by losing sex-determining genes. In particular, a low-density population and heterogamous mating could cause increased selection pressure from dioecy to hermaphrodite^[19]^.

## Types of plant sex chromosomes

The sex determination systems in plants are nominated based on karyotypes of the sex chromosomes, including the simple sex karyotypes and the complex sex karyotypes. The simple sex karyotypes consist of XY/XX, ZZ/ZW, XO/XX, and ZO/ZZ (Table 1), among which, the XY/XX system is the most common sex determination system, with XY appearing as male and XX appearing as female. Plant species with the XY sex-determination system include *Spinacia oleracea*^[20]^, *Silene latifolia*^[21]^, *Populus deltoides*^[22]^, etc. The complex sex karyotypes contain X_n_Y, XY_n_, and X_n_Yn (n: the number of sex chromosomes), such as *Botryococcus braunii* (X_n_Y), *Rumex acetosa* (XY_n_), and *Humulus lupulus* (X_n_Y_n_)^[23]^. Besides, some plants have sex chromosomes, their sexual phenotypes are not merely determined by sex chromosomes, but by the ratio of sex chromosomes and autosomes, such as hops (*H. lupulus*)^[24]^, date tree (*Phoenix dactylifera*)^[25]^ and *R. acetosa*^[26]^.

**Table 1:** Results of sex determination of representative plants.

Taxon	Species	Sex determination system	Sex-linked region or genes	Ortholog gene or family	Reference
Bryophyte	*Marchantia* *polymorpha*	XY	14 male-specific genes	—	[27,30]
Gymnosperm	*Ginkgo biloba*	ZW	*GbMADS18, Gb_15883,* *Gb_15884, Gb_15885,* *Gb_15886, Gb_28587*	*MADS-box (GbMADS18),* *RR12 (Gb_15883),* *RR2 (Gb_15884),* *ELF6 (Gb_15885),*	[30−33]
				*AtBAT1 (Gb_15886),*	
				*AGL8 (Gb_28587)*	
Angiosperm	*Fragaria virginiana*	ZW	*GMEW, RPP0W*	GDP-mannose 3,5- epimerase 2 (*GMEW*), 60S acidic ribosomal protein P0 (*RPP0W*)	[5, 34]
	*Spinacia oleracea*	XY	LG4 (66.98−69.72 cM and 75.48−92.96 cM)	—	[35,20]
	*Silene latifolia*	XY	*SlAP3, SlSTM, SlCUC*	*AP3 (SlAP3), STM (SlSTM), CUC1/CUC2* *(SlCUC)*	[36,37]
	*Phoenix dactylifera*	XY	*CYP703, GPAT3*	*—*	[38]
	*Actinidia chinensis* *A. deliciosa*	XY	*SyGl, FrBy*	*ARR24(SyGl),* *FAS1(FrBy)*	[39,40]
	*Asparagus officinalis*	XY	*SOFF, aspTDF1*	*DUF247 (SOFF),* *TDF1 (aspTDF1)*	[41,42]
	*Diospyros lotus,* *D. kaki*	XY	*MeGI, OGI*	*HB40 (MeGI)*	[42,43]
	*Cucumis melon*	—	*ACS7, ACS11,* *WIP1, CRC*	*—*	[43−46]
	*C. sativus*	—	*ACS7, ACS11,* *WIP1, CRC*	*—*	[43−46]
	*Carica papaya*	XY	*CpSVPL, CpSERK, CpCAF1AL*	—	[41,47−49]
	*Myrica rubra*	ZW	59 kb female- specific region on chromosome 8	—	[50]
	*Cannabis sativa*	XY	sex chromosomes	—	[51]
	*Vitis vinifera*	XY	*VviINP1, VviYABBY3*	*INP1 (VviINP1),*	[52]
				*YAB1 (VviYABBY3)*	
	*Populus deltoides,* *P. tremula, P. alba, P. trichocarpa, P. balsamifera,* *P. tomentosa*	XY, ZW	*FERR-R’, FERR,* *MmS, ARR17*	*ARR17*	[22,53,54]
	*Humulus lupulus*	XY	Gr09-M	*—*	[55]
	*Salix purpurea,*	ZW	RR	*RR9/ARR17*	
	*S. triandra*				[55,56]

Sex chromosomes can either be homomorphic or heteromorphic, although plant species with homomorphic sex chromosomes are more common in nature^[27]^. Heteromorphic sex chromosomes (also referred to as allosomes) can be distinguished by the differences in morphs, sizes, and pairing behavior from the autosomes, while homomorphic sex chromosomes are similar to the alternate sex chromatids^[28]^. The difference between homomorphic and heteromorphic sex chromosomes relates to degree of the genetic divergence (SNPs, inversions, or deletions) between the chromosomal pairs. Deletions of large parts of the Y or W chromosomes result in detectable Y and W heteromorphism, or even X0 (without X) or Z0 (without W) systems. Heteromorphic sex chromosomes were first detected by cytogenetic methods. Afterward, many of the old-established and highly degenerated sex chromosomes were discovered in heteromorphic sex chromosomes in plants^[29]^.

## Recombination suppression in plant sex chromosomes

It is a common scenario that homologous chromosomes can't recombine and pair, which is the driving force for the divergence of sex chromatids. Recombination suppression occurs within the male-specific region of the Y chromosome (MSY), the female-specific region of the W chromosome (FSW), as well as between X and Y (Z and W) chromosomes^[57]^. The recombination-suppressed region has a trend that continuously expands toward the pseudo-autosomal region (PAR)^[58]^, while XX (or ZZ) and PAR recombination maintains normal^[59]^. Recombination suppression also occurs between homomorphic sex chromosomes^[60]^.

Recombination suppression on sex chromosomes is normally caused by repetitive sequence accumulation, chromosomal inversions, chromosomal heterochromatinization, DNA methylation, etc. Accumulation of repetitive DNA sequences (including transposable elements and satellite DNA), can create a divergence between sex chromosomes. This divergence could cause morphological and molecular structural changes on incipient sex chromosomes, causing some male- or female-specific hetero-sequences to accumulate and spread on chromosomes^[3]^. The acquisition of sexually antagonistic alleles is also a primary driver of recombination suppression^[61]^. Antagonistic alleles will confer a fitness advantage that is beneficial for one sex and detrimental to the other, resulting in one allele being selected for retention^[62]^.

Recombination suppression regions occur on many plant sex chromosomes. Both sex chromosomes of *Actinidia chinensis* have a recombination mode, and the overall pseudo-autosomal region recombination rates in male parents were higher than those in females^[63]^; whereas in *H. lupulus*, the recombination suppression of females was estimated to be four times higher than that of males, with evidence that genetic distance of two shared markers linked to sexes was 3.7 cM in male, but 14.3 cM in female^[55]^. Recombination suppression prevents harmful mutations on sex chromosomes from being eliminated in time by chromosomal recombination suppression persists a long time, it will promote the genetic degeneration of the plant Y(W) chromosome to change the adaptability of Y(W) or X(Z) chromosome-linked genes^[64]^ , including the evolution of dosage compensation^[65]^. Genetic genomic degradation of a young Y chromosome in *Drosophila miranda*. Degeneration is mainly characterized by loss of gene composition, and function manifested by shortened Y chromosome in appearance^[66]^. There are two strong pieces of evidence: one is that the majority of YY genotypes in plants are inactive, and the other is that the X chromosome-linked gene *MROS3* was identified in *S. conoidea*, which had been degraded on the corresponding Y chromosome^[67]^.

## DNA methylation affects sex determination in plants

Epigenetic modifications affect gene expression without altering the DNA sequence and play a significant role in all aspects of plant development, physiology, and reproduction^[68]^. DNA methylation is a relatively conserved epigenetic modification that is important for gene regulation and genomic stability^[69]^. Cytosine methylation occurs at different stages of flower development in *Arabidopsis thaliana*^[70]^. In garden asparagus (*Asparagus officinalis*), the level of DNA methylation in female flowers is higher than that in male flowers at the same stages^[68]^. Differential cytosine methylation can affect the activity of the Y chromosome of dioecious plants. Hypermethylation occurs in the Y chromosome of *S. latifolia*, which has some impact on chromosomal heterochromatin and affects the evolution of the Y chromosome^[71]^.

In addition, the methylation status of sex-determining genes can alter sexual morphs of some plants. For example, sex in cultivated persimmon (*Diospyros kaki*) is determined by the epigenetic regulatory factor*.* The methylation level of *MeGI* can be accumulated and reset, leading to the sex of flowers changing on the branches of the offspring of this plant, yielding female or monoecious flowers; whereas hypermethylation of the *CmWIP1* gene in melon (*Cucumis melon*) results in the turnover of bisexual flowers into female flowers^[44, 72].^ Methylation can regulate the chromosomal structure to adjust the sex ratio according to the developmental signals of sex determinants. Differential methylation status of *PbRR9*, which is located in the sex-linked region (SLR) of *P. balsamifera*, was found in its promoter and the first intron. These two methylated regions triggered the sex differentiation of *P. balsamifera*^[53]^.

## Methods detecting the SLRs

The phenotypic and genetic studies of sex chromosome mutants show that sex chromosomes contain genes that regulate the specific development of male and female individuals or organs. Therefore, detecting SLRs are genome sites for studying recombination suppression, genetic degeneration, sex-biased expression, accumulation of repeat sequences, and time scales of heterochromatin formation. Three approaches are commonly adopted to detect SLRs. (1) Molecular marker approach, which is widely used for detecting the completely linked regions of genetically variable genes in non-model species. However, this method can only obtain the genetic distance of markers and roughly estimate the size of SLRs. (2) Sequence mapping approach, by sequencing and mapping the sequence reads to the reference genome, we can detect the most divergent chromosomal regions and identify the hemizygous Y and W regions. However, this method may have limited application for species with sex chromosomes containing only a few sex-determining genes and SNPs. (3) Genome-wide association analysis (GWSA) approach, which can detect fully sex-associate regions between the sexes^[73]^. The latter two approaches can determine the size of SLRs more precisely than the molecular marker approach, but with a higher presence of false positive signals. It is better to confine the SLRs first by the molecular marker approach, and then to characterize the details of SLRs by a joint adaptation of the latter two approaches.

## Recent progress in studying plant sex determination

The study of sex determination in dioecious species has long been a keen topic in plant reproduction biology, which is not only of theoretical importance but also of critical importance in plant breeding programs. With the rapid development of sequencing technology, more and more sex-determining genes in dioecious plants have been identified and cloned. The sex determination of Ginkgo (*Ginkgo biloba*) is a XY system, rather than that of the ZW system based on karyotype analysis^[31]^. By resequencing, reads of 100 individuals from the Ginkgo half-sibling pedigree, a whole male determination region in Ginkgo was identified. 3,647 SNPs that were significantly associated with sex differences identified by GWAS analysis. Besides, by mapping the resequencing reads of 100 individuals from the Ginkgo siblings to the reference genome (female Ginkgo tree), 48.5% of the 3,611 SNPs (SNPs continuously distributed on chromosome 2, the sex chromosome of Ginkgo) were heterozygous in males, while 3,043 loci of these 3,611 (84.3%) were homozygous in females. Combined with genetic markers, it was further determined that chromosome 2 was the sex chromosome of Ginkgo, and its 48−75 Mb interval was the SLR on the Y chromosome. The authors detected 200 genes (including four MADS-box genes and two *PPR* genes) that are specifically expressed in males. The levels of flavonoid compounds were also found to significantly differ between the sexes^[33]^.

The results of genome-wide association studies showed that diverse poplar species (including *P. tremula* and *P. trichocarpa*) carry partial duplicates of the ARABIDOPSIS RESPONSE REGULATOR 17 (*ARR17*), which contains sex-related SNPs in the male-specific region of the Y chromosome. Male-specific partial *ARR17* duplicates are arranged as inverted repeats. *ARR17* male-specific non-encoded transcripted a large number of sRNAs, especially 24-nt sRNAs, which could be pinpointed to two SLR regions. Bisulfite sequencing showed DNA methylation enrichment at the *ARR17* male region-specific site, indicating that *ARR17* is regulated by apparent silencing. CRISPR-Cas9-induced mutations demonstrate that *ARR17* functions as a sex switch, triggering female development when on and male development when off. Sequencing and *de nov*o assembly of a female *P. alba* tree yielded a W-chromosomal contig comprising three female-specific complete copies of *ARR17*, while absent on the Z chromosome. In conclusion, the sex-specific regulation of *ARR17* is conserved across the poplar genus and probably beyond^[54]^_._

Cucumber is monoecious and emerged as an excellent model system to explore sex determination. Sexual forms are primarily controlled by three alleles (*M*/*m*, *F*/*f*, and *A*/*a*)^[74−76]^. All genes, including *f* gene (*CsACS1*), *F* gene (*CsACS1G*)^[77]^, *M* gene (*CsACS2*)^[78]^ and *A* gene (*CsACS11*), participated in the plant hormone ethylene biosynthesis pathway^[45]^. Among them, the dominant *A* gene (androecious) controls carpel development, while recessive homozygous *aa* represents the all-male phenotype. The authors cloned the androecious gene *CsACS11* (a) through a positional cloning strategy. One-base deletion of *CsACS11* in the third exon causes premature termination of *CsACS11* translation. *CsACS11* was expressed in phloem cells connected to flowers programmed to become female. Mis-sense mutation of *CsACS11* results in all-male plants. *CmACS11* is thought to act upstream as a negative regulator of the expression of *CmWIP1*^[45]^. The carpel identity gene CRABS CLAW (CRC) was isolated from a female-to-male sex transition mutant in cucurbits. But the expression CRC was suppressed through histone deacetylation by the transcription factor *Cm*WIP1. *Cm*WIP1 can recruit the corepressor TOPLESS (TPL) and form to WIP1-TPL complex binds to the CRC promoter to inhibit its expression. Thereby promoting the development of male flowers. CmWIP1 promotes male flower development through interference with CRC function in floral meristem determinacy in the carpel primordia^[46]^.

Papaya (*Carica papaya*) is a trioecious species with three sex types. Female XX, male XY, and hermaphrodite XY^h^. Y^h^ chromosome of papaya diverged from its Y chromosome ancestor about 4,000 years ago^[79]^. More accumulation of DNA exsits in the male regions in Y chromosome of* C. papaya* compared to the corresponding X chromosomal region. The four specific heterochromatin furuncles in Y chromosomes explained that DNA methylation, together with heterochromatinization plays vital roles in the early evolution of sex chromosomes in papaya. The two insertions emerged in sex-determining regions of Y^h^ chromosome about 700 and 190 million years ago. A great quantity of retrotransposon exists in partial regions of HSY, causing the recombination suppression with X chromosome. When recombination suspends, Y chromosome starts to rearrange internally, and transportable elements accumulate during the preliminary stages of evolution, resulting in increased physical distance within its sex chromosome regions.

The sex determination of papaya is controlled by genetic and epigenetic regulators^[48]^. Liu et al. performed a comparative transcriptomic analysis of different sexes of floral buds with initiated reproductive organ primordia and identified 11 genes differentially expressed in the SLR of papaya and nine genes involved in stamen and carpel development^[49]^. Multiple transcription factors, epigenetic and phytohormone regulators (such as ABA and auxins) might be involved in the sex differentiation of papaya. Effects of methylation and hormone action may play important roles in sex dimorphism formation and sex chromosome evolution in papaya^[80]^. By comparing the bisulfite sequencing of genomic DNA from early-stage flowers of dioecious and gynodioecious papaya grown in two seasons (summer and spring), the seasonal methylated variances and dynamics among different papaya sex types were investigated. They observed the SDR of sex chromosome was hypermethylated, sex divergence did not greatly affect the chromosomal methylation landscapes of Y^h^ and Y^[80]^.

The areca palm (*Areca catechu* L.) has monoecious spadixes with the spatial separation of flower buds of different genders. Males on the apical side and females on the basal side had staggered blooming time, thus avoiding self-pollination. The high-quality reference genome of *A. catechu* was obtained by PacBio and HiC sequencing technologies with the value of 2.7 Gb, 16 pseudo-chromosomes and encoding 31,406 genes. Genes related to JA biosynthesis and signaling pathways had sex differentiation profiles *via* epigenetic modifications in *A. catechu* by ATAC and RNA-seq. In female flowers, the specific chromatin regulatory regions for genes related to JA synthesis and signal transduction are open, gene expression level consequently promoted and JA production increased. The opposite is true in male flowers. Furthermore, JA promotes the development of female flower organs by decreasing the expression of B-function genes, including AGL16, AP3, PIb and PIc. The sex-related region was located on pseudochromosome 15 by comparative genome analysis^[81]^.

Based on the experimental results on plant sex determination obtained thus far, sex separation of plants could either occur through the two-gene model or the one-gene model.

### Sex determination *via* two genes

Strong experimental evidence has been found in asparagus (*A. oﬃcinalis*)^[41]^ and kiwifruit (*A. deliciosa*)^[^^39^^]^, which support the two sex-determining gene model. In garden asparagus, its sex is found to be determined by *TDF1* and *SOFF*. By sequencing the whole genome of garden asparagus, Harkess et al.^[41]^ excavated the *TDF1* gene, which locates in the non-recombined Y-specific region. It encodes an R2R3MYB transcription factor. When knocking out the *TDF1* homologous gene in *Arabidopsis,* the plant exhibits a male-sterile phenotype. Subsequently, combined with mutagenesis screening and smFISH (single-molecule fluorescence *in situ* hybridization) technology, a Y-chromosome-specific suppressor of female function (*SOFF*) was identified. It inhibits the development of pistils and is necessary for anther growth. The SOFF protein contains a pfam-annotated unknown functional domain DUF247. A frameshift mutation exists in the DUF247 domain, causing the transition of XY male to hermaphrodite, which suggests *SOFF* is a female suppressor gene. The Y SLR containing *SOFF* and *TDF1* is a hemizygous segment, which is only present on the Y chromatid, but absent on the X chromatid. Sex of kiwifruit also occurs through the XY sex-determining system. The sex chromatid pair of kiwifruit are morphologically identical and are in the very early evolutionary stage of sex chromosomes, which contain a short SLR. By mapping the resequencing data of males and females to the reference sex chromosome, a female suppressor, *SyGI*, was identified, which inhibited female function by altering cellular patterns and disrupting the ability of stigmas to promote pollen tube growth. Subsequently, the authors cloned a male-specific expressed gene, namely *FrBy*^[42]^, which is a male promotor. The Y-specific *SyGI* and *FrBy* act as the SuF and M factors in Charlesworth's model^[13]^, respectively.

**Figure 3:**
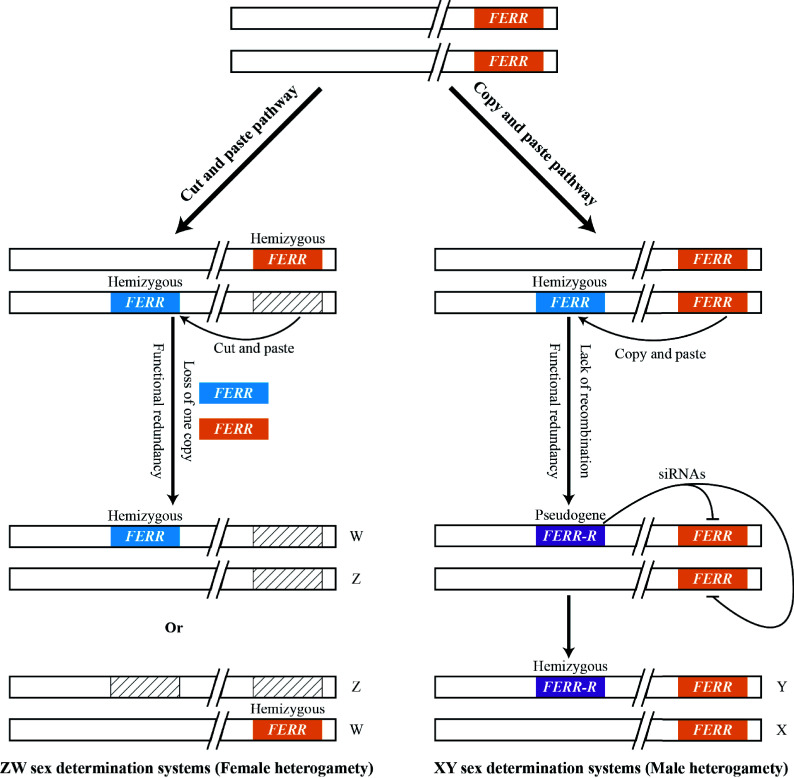
Two transposition pathways (copy and paste) leading to the emergence of XY and ZW sex determination systems in *Populus deltoides.*

The Salicaceae species are typically dioecious plants. *P. deltoides* belongs to the genus *Populus*, in which, two Y-specific genes, *FERR-R* and *MSL*, are found to trigger the sex determination of this species^[22]^. The *FERR-R* gene is duplicated from the *FERR* gene located in the pseudo-autosomal region (PAR) of the sex chromosomes. Overexpression of the *FERR* gene in *Arabidopsis* promotes pistil development but does not affect the stamen. *FERR-R* generates sRNAs, which guide DNA methylation of the *FERR* gene and also target the* FERR* transcripts, leading to silencing of *FERR* in males. While the *MSL* gene generates long non-coding RNAs (lncRNAs), overexpression of *MSL* in *Arabidopsis* promotes stamen development but does not affect the pistil. This study showed that the sex of *P. deltoides* related to expression of these two genes. However, knocking out the homologous gene of *FERR* in female *P. tremula* resulted in complete sex turnovers^[54]^, indicating sex differentiation of *P. tremula* may be triggered by only one gene. The gender locus was mapped to different positions in some of the Salicaceae species, and both XY and ZW sex determination systems were reported for plants in the Salicaceae family^[82]^. In Salicaceae species, the SLR was positioned at the telomeric or the centromeric region of chromosome 19 in a variety of poplars. While in willows, the SLR was consistently mapped to the centromeric region of chromosome 15^[56]^, except for *Salix nigra*, whose SLR was positioned on chromosome 7^[83]^. Both the XY and ZW sex-determination systems have been observed in different species of Salicaceae. However, a common finding is that one or more duplicates of the *RR* gene determine the femaleness (namely *FERR* in *P. deltoides* and *ARR17* in *P. tremula*) present in the SLR of different Salicaceae species^[54]^. In the SLR of *Salix chaenomeloides* and *S. Arbutifolia* locates intact and/or partial homologs of a type A cytokinin response regulator (RR) gene. Ancestors of willows had a sex determination system similar to that of *S. chaenomeloides* on chr 7, while accumulation of deleterious mutations on Y-SDR make the RR partial duplicates translocate from chr 7 to chr 15 in the first turnover event, maintaining an XY system. Meanwhile, the SDR of *S. purpurea* accumulates many intact RR genes, manifesting expression preference, causing the transition from a XY system to a ZW system, concurrent with the model of 'deleterious mutation load' and 'sexually antagonistic selection'^[84]^. The result suggests that the flexible gender locus positions and the reversible sex determination systems (XY and ZW) among different species might relate to transpositions of the *RR* gene determining femaleness. Transpositions of the *RR* gene either occurred through the 'copy and paste' pathway or through the 'delete and paste' pathway. The 'copy and paste' pathway would produce an additional copy of redundant *RR* genes, subsequently, functional redundancy would lead to dysfunction of the duplicate. The hypothesis is that in the 'copy and paste' transposition pathway, the duplicate is a hemizygous copy, lack of recombination would make the duplicate more vulnerable to becoming a pseudogene. By contrast, the original allelic copies of the gene are present on both chromatids, and recombination of the sister chromatids will help maintain function of the original copy. Such as in *P. deltoides*, dysfunction of the duplicate resulted in a Y-specific pseudogene (*FERR-R*) that generated sRNAs targeting the original *RR* gene (*FERR*, allelic presence on both X and Y). This pathway would generate heterogamic males, leading to the emergence of XY sex determination system in the affected species. Whereas the 'delete and paste' transposition would result in two copies of unlinked hemizygous *RR* genes. Subsequently, loss of one of the duplicates would generate heterogamic females, leading to the emergence of ZW sex determination system in the affected species (Fig. 3). Additional studies need to be carried out in other Salicaceae species to better understand the sex determination in these taxa of plants. Nevertheless, the Salicaceae species provide desirable systems to understand the occurrence of the XY and ZW sex determination systems in closely related plants.

Apart from the aforementioned plant, the wild grape (*Vitis vinifera*) is a dioecious plant with XY sex-determining system, while many grape varieties exhibit both dioecious and hermaphroditic sex morphs. In the grape variety, Cabernet sauvignon, the* VviINP1* gene was identified as a candidate maleness promotor, which expressed and functioned in all sex morphs, but in females, the expression level is higher than in males and hermaphrodites. An 8-bp deletion was detected on the *VviINP1* gene in the females, resulting in the frameshift mutation and early termination of the encoded protein, causing loss of gene function. By contrast, all males carried a functional copy of *VviINP1*. Another gene associated with female fertility is the *VviYABBY3* gene^[52]^. In* A. thaliana*, its homologous gene was revealed to be involved in carpel development. Thus, current data showed that sex of dioecious grape might be determined *via* at least two genes. The VviPLATZ1 transcription factor is a key candidate female flower morphology factor that localizes to the Vitis SEX-DETERMINING REGION. After generating multiple CRISPR/Cas9 gene-edited alleles of VviPLATZ1 in a rapid cycling hermaphrodite genotype, phenotype analysis shows that individuals harboring gene-edited alleles of VviPLATZ1 lines produce flowers with reflex stamens, which means Vvi-PLATZ1 is a key regulator of female flower formation in grape-vine^[85]^.

### Sex determination *via* one gene

Mutations of sex genes in promoting male or inhibiting female will significantly reduce the fitness of female-related functions. In other words, there is a genetic conflict between male-promoting genes and female-promoting genes^[86]^, which enhances the linkage inheritance of multiple male gain genes. However, if the fitness of the female-related functions is free from the male-promoting or female-inhibiting mutations, and stays favorable enough for the male individuals, such mutations can be retained and spread in the population even if they are not linked to female-related genes, thus resulting in a single gene sex-determination system^[87]^. A single gene can also determine the sex of dioecious plants such as *D. lotus*^[88]^, *P. balsamifera*, *P. trichocarpa*^[54]^, and *M. polymorpha*^[30]^.

The genus *Diospyros* contains approximately 475 species, which are all dioecious^[89]^. A Y-specific sex determination gene *OGI* was uncovered in males of *D.* species. The male sex was found to be mediated by the *OGI* gene located in a Y-specific segment, which repressed the *MeGI* gene that is located on the autosome. Over-expressed *MeGI* in *A. thaliana* and *Nicotiana tabacum* inhibited stamen development. The *OGI* is a noncoding gene duplicated from *MeGI*. It produces siRNAs targeting *MeGI*, thereby blocking the expression of *MeGI* in males and releasing the inhibition on stamens. In females, *MeGI* expresses normally due to the absence of the *OGI* gene.

Sex chromosome was also observed in *M. polymorpha*, leading to the separation of male and female plants. The *Feminizer* gene, a transcription factor on the U chromosome of *M. polymorpha* was identified as the sex-determining gene encoding plant-specific basic pentacysteine. The *Feminizer* triggered female differentiation by directly regulating *FEMALE GAMETOPHYTE MYB* and *SUPPRESSOR OF FEMINIZATION* genes on the autosome. *Feminizer* also plays a role in reproductive induction, consistent with the function of its gametophyte homolog on the V chromosome, suggesting the ancestral sex determination mechanism during the evolution of a haploid sex chromosome system^[30]^.

## The evolutionary pathways of unisexual flowers

Sex-determining genes initiate sex differentiation in reproductive organs, while sex-differentiation genes take effect after the formation of the floral organ primordium^[90]^. The plant sexes can be distinguished based on the morphology of floral organs. Development and formation of floral organs can be generally elucidated by the ABCDE model, which is relatively conserved in the plant kingdom^[91]^. To our knowledge, most genes regulating floral organ development in hermaphrodite plants are homologous to that in *Arabidopsis*. In nature, different sexual morphs exist during the transition of hermaphroditic flowers into unisexual flowers. Most unisexual flowers have a bisexual stage in the early stage of development, whereas both pistil and stamen are present in a single flower, but one of the alternate sex organs aborts afterward. Two main ontogenic pathways underlying functionally unisexual ﬂowers have been proposed, i.e., the 'unisexual by abortion' pathway (type I) and the 'unisexual from inception' pathway (type II)^[19]^. Sex-determining genes act as primary regulators and interact with B, C, or D MADS-box transcription factors. In terms of the 'unisexual by abortion' pathway (type I), the abortion of unisexual flowers could occur at stage 1 (in the early development stage of stamens or pistils), stage 2 (pre-meiosis of microspore or megaspore mother cells), or stage 3 (post-meiosis)^[10]^. In the type I unisexual flowers, flowers bear both stamen and pistil, but one of the sex organs is infertile, such as *A. oﬃcinalis*^[41]^, *A. deliciosa*^[39]^, and so on. Contrary to the type I unisexual flowers, sex organ abortion of Type II unisexual flowers occurs before the formation of floral primordia, resulting in flowers bearing either only stamen or only pistil, such as that of *Quercus variflora*^[92]^, *P. tomentosa*^[93]^ and so on.

## Regulation of plant hormones

Once plant sexual differentiation initiates, different male and female flowers gradually form^[49]^. The sex phenotype of plants has certain plasticity; some substances like phytokinin can change the expression of the original program, thus controlling gender expression. Hormone levels can be indirectly regulated by sex-determining genes, and hormone-induced differentiation programs are then used to regulate sex phenotype formation. The sex-regulatory mechanism of related hormones has been reported in multiple plants such as *C. melo*^[45]^*,* maize^[94]^, and* Actinidia*^[39]^. The changes in endogenous hormone levels could affect plant sex differentiation, the ratio of male to female flowers induced by the addition of exogenous hormones.

Some sex-determining genes involved in cytokinin and ethylene-related plant hormone regulatory pathways have been reported in angiosperms. For example, SDGs (or candidate SDGs) in date palm, kiwi, grape, and poplar are associated with cytokinins, while ETO1 and FIG RAN1(candidate sex-determining genes) in grape are associated with ethylene signaling pathways. Furthermore, several genes such as hormone-responsive genes, including ARABIDOPSIS RESPONSE REGULATOR 17 (ARR17), Lonely-Guy (LOG1)^[38]^, 1-aminocyclopropane-1-carboxylic acid synthase 11 (ACS11)^[45]^ {Picq, 2014 #190; Boualem, 2015 #191} have been reported to regulate sex determination and floral morphogenesis. Further molecular mechanisms of sex differentiation by these hormones need to be investigated.

## Conclusions and perspectives

Sex dimorphisms and sex ratio in a population of dioecious plants are directly associated with the economic values of agricultural crops and forest trees. For cash crops, such as kiwifruit (*A. chinensis*), cucumber (*C. sativus*), and pecan (*Carya illinoinensis*), the increase in female plants would improve the yield. For gingko trees, the males usually perform better than the females, increase males in the population would yield a higher economic value. For poplars and willows, when female trees arrive sexually mature ages, they produce heavy catkins, causing severe air-borne pollution, thus the females are unfavorable in forestation. As a result, identification of the sexes of dioecious plants is influential. However, sexes of dioecious plants can't be distinguished before flowering. Uncovering the molecular mechanism underlying plant sexes enable us to develop gender-identification technology based on gender determining genes or gender-specific sequences. With such technology, we can precisely identify the sexes of plants when they are in the juvenile stage.

Dioecy has evolved many times repetitively and independently in different phyla of botany. In addition, sex organ abortion occurs at different developmental stages of unisexual flowers. Multiple lines of evidence suggest that the separation of sexes in different plants may involve different genes, which regulate the upstream or downstream pathway of floral development. This proposal has been evident by the most advanced studies in this area. The uncovered plant sex-determining genes also offer us a good chance to characterize the SLRs containing the sex-determining genes, thereby elucidating the evolution of plant sex chromosomes. However, such research isrelatively scarce. Although great achievements have been made in cloning the sex-determining genes in dioecious plants in the recent decade, only ~0.3% of these species have highly sequenced genomes data, and limited plant species have been well studied thus far. It is necessary to increase the number of high-quality genome assemblies of different phylogenetic breadth of dioecious species. Pan-genome should be encompassed within species in the future. It's better to understand some non-coding sequences, like small RNAs' function in sex-specific development and sex determination. Moreover, advanced technologies, such as CRISPR will likely improve the functional validations for more species.

The details of the regulatory networks of sex-determining genes and the genetic mechanisms underlying plant sex liability and the occurrence of intermediate sex morphs remain largely unknown. More interdisciplinary work and cytological analyses are needed to discover a wealth of rules taking part in the complex and dynamic regions of the sex chromosomes in plants. With the development of sequencing technologies, together with the established analytical tools, research progress in this area would be greatly accelerated.
